# Cost-effectiveness of a potential Zika vaccine candidate: a case study for Colombia

**DOI:** 10.1186/s12916-018-1091-x

**Published:** 2018-07-03

**Authors:** Affan Shoukat, Thomas Vilches, Seyed M. Moghadas

**Affiliations:** 1 Agent-Based Modelling Laboratory, York University, Toronto, ON M3J 1P3 Canada; 2 Department of Biostatistics, Institute of Biosciences, São Paulo State University (UNESP), Botucatu, SP 18618-689 Brazil

**Keywords:** Zika, Microcephaly, Vaccination, Agent-based modeling, Cost-effectiveness

## Abstract

**Background:**

A number of Zika vaccine platforms are currently being investigated, some of which have entered clinical trials. We sought to evaluate the cost-effectiveness of a potential Zika vaccine candidate under the WHO Vaccine Target Product Profile for outbreak response, prioritizing women of reproductive age to prevent microcephaly and other neurological disorders.

**Methods:**

Using an agent-based simulation model of ZIKV transmission dynamics in a Colombian population setting, we conducted cost-effectiveness analysis with and without pre-existing herd immunity. The model was parameterized with estimates associated with ZIKV infection, risks of microcephaly in different trimesters, direct medical costs, and vaccination costs. We assumed that a single dose of vaccine provides a protection efficacy in the range 60% to 90% against infection. Cost-effectiveness analysis was conducted from a government perspective.

**Results:**

Under a favorable scenario when the reproduction number is relatively low (*R*_0_ = 2.2) and the relative transmissibility of asymptomatic infection is 10% compared with symptomatic infection, a vaccine is cost-saving (with negative incremental cost-effective ratio; ICER) for vaccination costs up to US$6 per individual without herd immunity, and up to US$4 per individual with 8% herd immunity. For positive ICER values, vaccination is highly cost-effective for vaccination costs up to US$10 (US$7) in the respective scenarios with the willingness-to-pay of US$6610 per disability-adjusted life-year, corresponding to the average per capita GDP of Colombia between 2013 and 2017. Our results indicate that the effect of other control measures targeted to reduce ZIKV transmission decreases the range of vaccination costs for cost-effectiveness due to reduced returns of vaccine-induced herd immunity. In all scenarios investigated, the median reduction of microcephaly exceeded 64% with vaccination.

**Conclusions:**

Our study suggests that a Zika vaccine with protection efficacy as low as 60% could significantly reduce the incidence of microcephaly. From a government perspective, Zika vaccination is highly cost-effective, and even cost-saving in Colombia if vaccination costs per individual is sufficiently low. Efficacy data from clinical trials and number of vaccine doses will be important requirements in future studies to refine our estimates, and conduct similar studies in other at-risk populations.

**Electronic supplementary material:**

The online version of this article (10.1186/s12916-018-1091-x) contains supplementary material, which is available to authorized users.

## Background

In November 2016, following the decline of Zika virus (ZIKV) outbreaks reported in 69 countries and territories [1], the World Health Organization (WHO) ended its declaration of ZIKV spread as a “*public health emergency of international concern*” [2]. However, sporadic cases of ZIKV infection have occurred [3], and the threat of large outbreaks continues to exist in the absence of countermeasures such as vaccination or prophylactic drugs, especially in susceptible populations where the primary transmitting vector (i.e., *Aedes aegypti* mosquito) is endemic [4]. Although vector-control programs can mitigate the impact of disease, ZIKV still remains an important public health concern due to its potential to cause severe outcomes and long-term sequelae, including microcephaly with brain abnormalities and neurological disorders in infants, and Guillain–Barré syndrome (GBS) in adults [5–7].

Previous studies indicate that a significant portion (up to 80%) of ZIKV-infected cases experience asymptomatic form of disease, yet are still capable of contributing to virus transmission [8]. There is also evidence that ZIKV can be transmitted through sexual encounter [9–11]. Furthermore, congenital ZIKV syndrome has been reported to occur in the same proportion of women with asymptomatic as symptomatic ZIKV infection during pregnancy [12]. These considerations instigated global efforts for the development of a safe and effective Zika vaccine. Currently, there are a number of vaccine candidates being investigated using a variety of vaccine platforms [13], including purified inactivated, live attenuated, viral-vectored, virus-like particles, recombinant subunit, DNA, self-replicating RNA, and mRNA [13, 14]. Experience with the development of other flavivirus vaccines suggests that generating a preventive Zika vaccine should be feasible [15, 16]. However, the cost-effectiveness of a vaccine candidate will be a major factor in decisions regarding the implementation and strategic use of vaccines in immunization programs.

In this study, we sought to investigate the cost-effectiveness of a potential Zika vaccine candidate, taking into account the WHO vaccine prioritization of women of reproductive age [17], including pregnant women, to prevent prenatal ZIKV infection and microcephaly as well as other severe brain anomalies. For this investigation, we considered the ‘outbreak response’ scenario prioritized in the WHO/UNICEF ZIKV vaccine target product profile, and employed an agent-based computational model to simulate disease dynamics and derive outcomes for cost-effectiveness analysis. We performed this analysis using parameter estimates extracted from published studies with a plausible range of costs for vaccine administration.

## Methods

We extended a previously established agent-based computational model of Zika infection dynamics as the basic framework [18] to include vaccination and Zika-associated congenital microcephaly during pregnancy. The comprehensive structure of the model encapsulates age-dependent individual attributes and population heterogeneities, and simulates disease spread in humans through vector (i.e., mosquitoes) and sexual transmission (Additional file 1). The model was parameterized with a population demographic distribution similar to that of Colombia, in which the health of every individual is characterized by several epidemiological statuses, including susceptible, infected and incubating, infectious and asymptomatic, infectious and symptomatic, and recovered. In the chain of ZIKV transmission, mosquitoes exhibit the statuses of susceptible, infected and incubating, and infectious. Infected mosquitoes remain infectious for their entire lifespan. Our analysis was conducted for an epidemic outbreak starting during a high-temperature season.

### Disease outcomes

There is evidence that associates the risk of microcephaly in infants to Zika infection in all trimesters of pregnancy [19], although the risk is significantly higher in the first and second trimesters [12, 20]. Previous studies, considering possible overreporting, have quantified the risk of developing microcephaly in both symptomatic and asymptomatic pregnant women [21, 22]. We considered the associated risks in a probabilistic approach to determine the microcephaly outcome in pregnant women at the time of infection. Infants with microcephaly who survive their first year of life were assumed to have significantly lower life expectancy [23, 24]. We also considered the effect of neurological and behavioral deficits due to microcephaly, leading to an impaired quality of life, quantified by disability weights provided in the Global Burden of Disease study [25]. In addition to neurological microcephaly, we considered the risk of developing GBS in ZIKV-infected individuals [26].

### Vaccination dynamics

Based on the WHO recommendations for vaccine prioritization [17], we implemented vaccination in the model for women between 15 and 49 years of age. We also considered vaccination of other individuals in the population between 9 and 60 years of age in order to reduce the risk of disease transmission to pregnant women. Vaccine-induced immunity reduced the risk of infection based on the protection efficacy sampled for each vaccinated individual. We assumed that ZIKV infection following vaccination (if it occurred) was asymptomatic without clinical manifestation. Naturally acquired immunity was assumed to provide full protection for a sufficiently long period of time, so that the risk of re-infection within the same epidemic season was eliminated.

### Parameterization

The baseline values and ranges of disease parameters in the model are rigorously described in a previous study [18], and are summarized in Table 1. This parameterization is based on the estimates of the mean reproduction number of *R*_0_ = 2.2 (95% CI 1.9–2.8) for Antioquia, Colombia [27], and the mean attack rate of 8% (95% CI 4% and 26%) [18, 28]. The risk of ZIKV-infected microcephaly during the first trimester was sampled in the range 5% to 14% [12, 20, 21]. This risk was reduced during the second and third trimester, and was sampled in the range 3% to 5% [12, 20]. The risk for developing GBS was between 0.025% and 0.06% for both symptomatic and asymptomatic individuals [26]. All ZIKV-infected individuals with clinical symptoms incurred short- and long-term direct medical costs, depending on disease outcomes. Short-term costs were associated with a physician visit (US$65) [29] and diagnostic microcephaly test (US$150) for pregnant women [30]. For microcephaly and GBS, we considered lifetime direct medical costs of US$91,925 and US$29,027, respectively [24, 30], which included hospitalization, treatment, and other associated medical costs.

**Table 1:** Parameter values and their associated ranges used for simulations and cost-effectiveness analysis

Parameter description	Baseline value (range)	Source
Transmission probability for infection	Baseline for *R*_0_ = 2.2	[27]
Human to mosquito	0.2851 to 0.3947 depending on the assumed relative transmissibility of asymptomatic infection compared to symptomatic infection from 0.9 down to 0.1	Transmissibility was estimated by calibrating the model to the basic reproduction number in the range 1.9–2.8 [18]
Mosquito to human	Assumed the same as human to mosquito	
Relative transmissibility of asymptomatic infection	0.1–0.9	[18]
Human infection parameters		
Intrinsic incubation period	Mean: 5.7 days (Lognormal); shape = 1.72; scale = 0.21	[37, 38]
Infectious period	Mean: 4.7 days (Lognormal); shape = 1.54; scale = 0.12	[18, 39]
Risk of infection through sexual encounter	1–5%	[18]
Fraction of infected cases experiencing asymptomatic infection	40–80%	[7, 8]
Risk of Guillain–Barré Syndrome (GBS)	0.025–0.06%	[26]
Mosquito lifespan and infection parameters		
Seasonal lifespan determined by a hazard function (Additional file 1)	Mean for high temperature season: 19.6 days Mean for low temperature season: 11.2 days	[18]
Extrinsic incubation period	Mean: 10 days (Lognormal); shape = 2.28; scale = 0.21	[40]
Number of mosquito bites	Determined by Poisson sampling with the mean of half-life for each mosquito	[18]
Risk of microcephaly		
First trimester (ends at 97 days of pregnancy)	5–14%	[12, 20, 21]
Second and third trimester	3–5%	
Life expectancy		
Without microcephaly	70 years	[24]
With microcephaly	35 years	
Probability of survival past first year of life for infants with microcephaly	0.798	[23]
Pre-existing level of herd immunity		
From previous outbreaks	8% (2.2–11%)	[18, 28]
Vaccination coverage		
Non-pregnant women from 15 to 49 years of age	60%	Assumed
Pregnant women	80%	
Other individuals from 9 to 60 years of age	10%	
Vaccine efficacy		
Preventing infection	60–90%	Assumed; sampled for each vaccinated individual
Costs		
Direct medical costs of microcephaly	US$91,925 per lifetime	[24, 30]
Direct medical costs of GBS	US$29,027 per lifetime	
Costs of physician visit for symptomatic cases	US$65	[29]
Zika test for symptomatic pregnant women	US$150	[30]
Vaccine costs per individual (includes dose, transportation, administration, wastage)	US$2–$50	Assumed [32]
Cost-effectiveness rates		
Disability weight for microcephaly	0.16	[25]
Annual discount rate	3%	Assumed

For vaccine implementation at the onset of simulations, we assumed a conservative vaccination coverage of 60% for non-pregnant women of reproductive age. The coverage for pregnant women in the same age group was set to 80% throughout the simulations. The vaccine coverage for other individuals between 9 and 60 years of age was set to 10%. While some ZIKV vaccine candidates have entered phase 1 clinical trials, there is currently no data available to indicate the level of vaccine-induced protection and the number of vaccine doses required. We therefore assumed that a single vaccine dose provides a protection efficacy in the range 60% to 90% against infection, which was sampled for each vaccinated individual and implemented as a reduction factor in disease transmission.

### Transmissibility

Quantification of the relative transmissibility of ZIKV asymptomatic infection compared to symptomatic infection is currently lacking. We therefore considered two scenarios with transmission factors of 0.1 (low) and 0.9 (high) to quantify this relative transmissibility [18]. Furthermore, the contribution of ZIKV symptomatic infection to the overall disease spread has not been estimated. In the absence of such estimates, we also considered two scenarios with reduction factors of 0.1 and 0.5 for symptomatic transmission to account for decreased mobility and lower exposure to mosquito bites through full clothing, mosquito repellents, or possible isolation during symptomatic infection [18]. The risk of sexual transmission was sampled for each encounter in the range of 1% to 5% [18].

### Cost-effectiveness analysis

We conducted the cost-effectiveness analysis from a government perspective and included only direct medical costs. The health impact of microcephaly and GBS to an individual’s quality of life was captured by disability-adjusted life years (DALYs), as recommended in the 1996 Global Burden of Disease study [31]. Based on the estimates for other flavivirus vaccines, we considered a range of US$2 to US$50 for vaccination costs per individual [32], including vaccine dose, administration, and 3% wastage. For a given price, the incremental cost-effectiveness ratio (ICER) of 5000 simulation runs was calculated using the formula:

$$ \mathrm{ICER}=\frac{{\mathrm{Cost}}_{\mathrm{Vaccination}}-{\mathrm{Cost}}_{\mathrm{No}\ \mathrm{Vaccination}}}{-\left({\mathrm{DALY}}_{\mathrm{Vaccination}}-{\mathrm{DALY}}_{\mathrm{No}\ \mathrm{Vaccination}}\right)} $$

We calculated the average ICER values and the associated 95% confidence interval using a non-parametric bootstrap method of 2000 replicates, and constructed the cost-effectiveness plane and acceptability probabilities to offer a visual representation of the joint distribution of costs and benefits. All costs were reported in 2017 US dollars. A discount rate of 3% was applied to both the costs and DALY calculations to consider preference for present value.

## Results

We ran 5000 independent Monte Carlo simulations of ZIKV infection dynamics with a scaled-down population of 10,000 individuals. Each simulation was seeded with a single ZIKV latent case and run for a time horizon of 1 year (Additional file 1: Figures S3–S6). Disease outcomes and vaccination throughout each simulation were recorded and used to calculate ICER values and cost-effectiveness probability.

### Vaccine cost-effectiveness

**Fig. 1:**
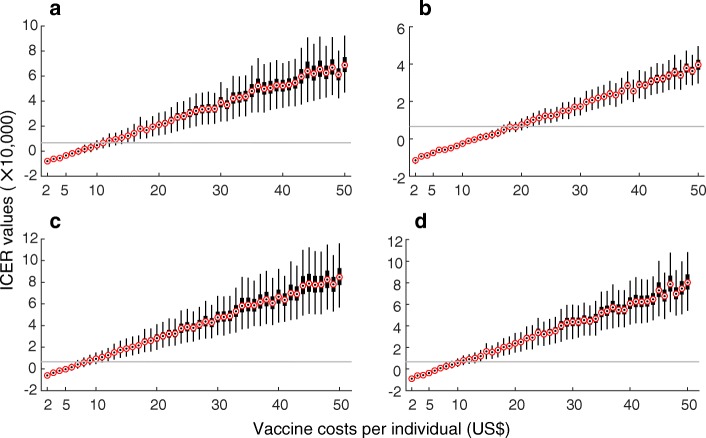
Boxplots for ICER values obtained using bootstrap method for a range of VCPI with *R*_0_=2.2. Subplots correspond to the scenarios without pre-existing immunity (**a**, **b**), and with an average of 8% pre-existingimmunity (**c**, **d**) in the population. The relative transmissibility of asymptomatic infection was set to 10% (**a**, **c**) and 90% (**b**, **d**). Solid (grey) line represents the willingness-to-pay threshold corresponding to the averageof per capita GDP of Colombia between 2013 and 2017

**Fig. 2:**
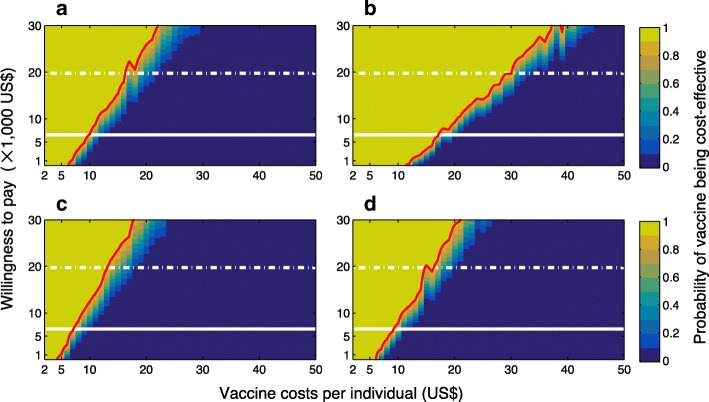
Probabilities of vaccine being cost-effective for a range of VCPI and willingness-to-pay, with *R*_0_ =2.2. Subplots correspond to the scenarios without pre-existing immunity (**a**, **b**), and with an average of 8% pre-existingimmunity (**c**, **d**) in the population. The relative transmissibility of asymptomatic infection was set to 10% (a,c) and 90% (b,d). Solid line represents the willingness-to-pay threshold corresponding to the average of per capita GDP of Colombia between 2013 and 2017. Dashed line represents three times the average of per capita GDP of Colombia. The red curve represents the 90% probability of vaccine being cost-effective for a given VCPI

We first considered *R*_0_ = 2.2 as the mean of a previously estimated range, which also lies within the range of estimates reported in previous studies for ZIKV spread in Latin and South America [27, 28]. With a relatively low reduction (10% on average) of transmission from ZIKV symptomatic infection, the ICER values for a range of vaccine costs per individual were calculated (Fig. 1). In a fully susceptible population, with a low (10%) relative transmissibility of asymptomatic infection (Fig. 1a), the ICER values and their associated ranges remained negative for 100% of simulation results when vaccination costs per individual (VCPI) were US$6 or less. These results suggest that the vaccine is cost-saving regardless of the thresholds of willingness-to-pay (Fig. 2a).

For VCPI with positive ICER values (Fig. 1a), we considered a range of willingness-to-pay values. At the conservative threshold of US$6610 per DALY averted, corresponding to the average per capita GDP of Colombia from 2013 to 2017, the ZIKV vaccine was highly cost-effective with a probability of at least 90% at a VCPI of US$10 or less (Fig. 2a). Increasing the threshold to US$19,832 (three times the average GDP) [32], our results suggest that vaccination would still be cost-effective for VCPI up to US$16. The probability of cost-effectiveness was sensitive to VCPI and decreased sharply from 90% to below 10% with a marginal increase in the VCPI.

When the transmissibility of asymptomatic infection was relatively high (90%), then vaccination was cost-saving for VCPI up to US$12, as suggested by the negative ICER values (Fig. 1b). For positive ICER values, vaccination was highly cost-effective at US$6610 willingness-to-pay per DALY averted at a VCPI of US$16 or less (Fig. 2b). At three times the GDP threshold of willingness-to-pay (US$19,832), vaccination was still cost-effective for VCPI up to US$29.

We investigated similar scenarios in the presence of pre-existing immunity as a result of previous outbreaks. We used estimates of attack rates with the mean of 8% (95% CI 4–26%) [18, 28] to account for herd immunity in the population. When the relative transmissibility of asymptomatic infection was low (10%), the ICER values and their associated ranges were negative for VCPI up to US$4 (Fig. 1c). For positive ICER values, vaccination remained highly cost-effective (with a probability of at least 90%) at the US$6610 threshold of willingness-to-pay per DALY averted when the VCPI did not exceed US$7 (Fig. 2c). At the threshold of three times the average GDP, vaccination was still cost-effective for VCPI up to US$13. With the same level of herd immunity, but a higher relative transmissibility of asymptomatic infection (90%), vaccination was cost-saving (with negative ICER values) for VCPI up to US$6 (Fig. 1d). When ICER values were positive, vaccination was highly cost-effective at a VCPI of US$8 or less, and cost-effective at a VCPI of US$14 or less at the willingness-to-pay thresholds of US$6610 and US$19,832, respectively (Fig. 2d).

**Fig. 3:**
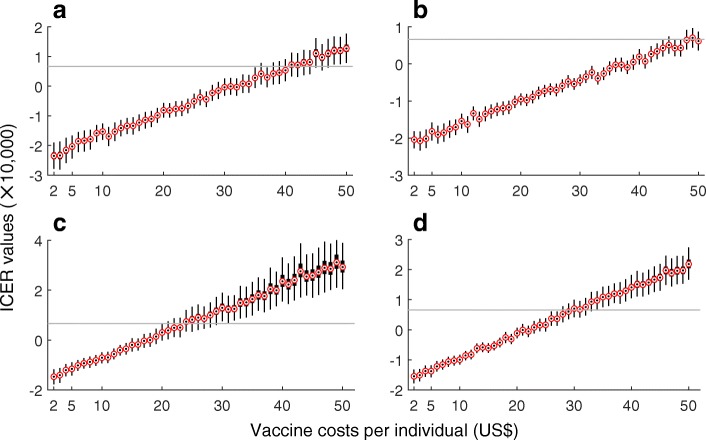
Boxplots for ICER values obtained using bootstrap method for a range of VCPI with *R*_0_ =2.8. Subplots correspond to the scenarios without pre-existing immunity (**a**, **b**), and with an average of 8% pre-existing immunity (**c**, **d**) in the population. The relative transmissibility of asymptomatic infection was set to 10% (**a**, **c**) and 90% (**b**, **d**). Solid (grey) line represents the willingness-to-pay threshold corresponding to the average of per capita GDP of Colombia between 2013 and 2017

**Fig. 4:**
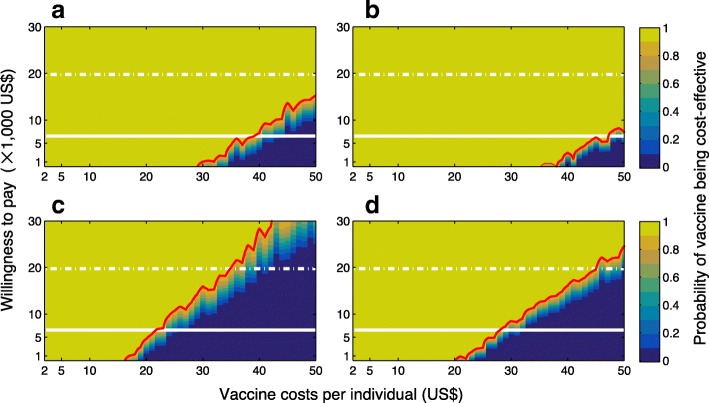
Probabilities of vaccine being cost-effective for a range of VCPI and willingness-to-pay, with *R*_0_ =2.8. Subplots correspond to the scenarios without pre-existing immunity (**a**, **b**), and with an average of 8% pre-existingimmunity (**c**, **d**) in the population. The relative transmissibility of asymptomatic infection was set to 10% (**a**,**c**) and 90% (**b**, **d**). Solid line represents the willingness-to-pay threshold corresponding to the average of percapita GDP of Colombia between 2013 and 2017. Dashed line represents three times the average of per capita GDP of Colombia. The red curve represents the 90% probability of vaccine being cost-effective for a given VCPI

In order to evaluate the vaccine cost-effectiveness in a population setting with a higher transmissibility, we considered the corresponding scenarios with *R*_0_ = 2.8. Compared to the case of *R*_0_ = 2.2, Figs. 3 and 4 indicate that vaccination is highly cost-effective for a larger range of VCPI, in particular when the reduction of transmission from ZIKV symptomatic infection is relatively low (10% on average). Table 2 summarizes simulation outcomes for VCPI in each scenario.

**Table 2:** Upper range of vaccination costs per individual (US dollar) for a Zika vaccine candidate to be cost-saving (ICER < 0), highly cost-effective (WTP of per capita GDP) or cost-effective (WTP of three times per capita GDP)

	0% herd immunity						8% herd immunity					
RTA	10%			90%			10%			90%		
		WTP			WTP			WTP			WTP	
*R*_0_	ICER < 0	$6610	$19,832	ICER < 0	$6610	$19,832	ICER < 0	$6610	$19,832	ICER < 0	$6610	$19,832
2.2	$6	$10	$16	$12	$16	$29	$4	$7	$13	$6	$8	$14
2.8	$29	$38	$53	$35	$45	$66	$16	$22	$35	$20	$27	$45

*ICER* incremental cost-effectiveness ratio, *RTA* relative transmissibility of asymptomatic infection, *WTP* willingness-to-pay

As the contribution of ZIKV symptomatic infection to disease spread decreases (e.g., reduction of 50%), a lower VCPI and a higher willingness-to-pay were required for the vaccine to be cost-effective (Additional file 1). We observed similar trends when the corresponding scenarios were simulated with an average of 8% pre-existing level of herd immunity in the population.

### Effect of vaccination on microcephaly

**Fig. 5:**
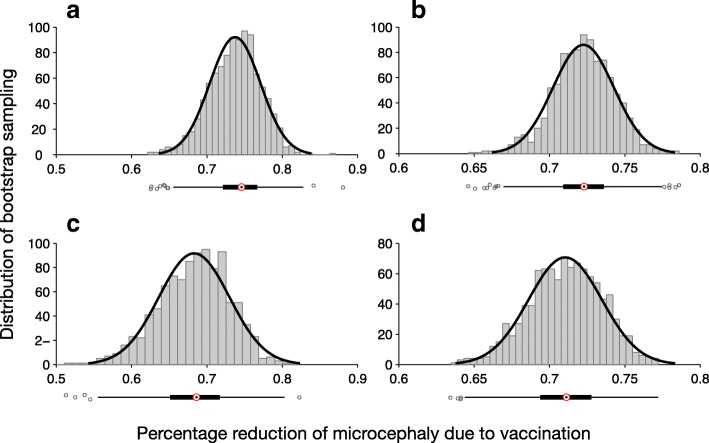
Distribution of percentage reduction of microcephaly obtained using bootstrap method, with *R*_0_ =2.2. Subplots correspond to the scenarios without pre-existing immunity (**a**, **b**), and with an average of 8% pre-existingimmunity (**c**, **d**) in the population. The relative transmissibility of asymptomatic infection was set to 10% (**a** ,**c**) and 90% (**b**, **d**). The median percentage reduction is (**a**) 0·739 (IQR: 0·715 – 0·759); (**b**) 0·723 (IQR: 0·709-0·736); (**c**) 0·687 (IQR: 0·652 – 0·717); (**d**) 0·711 (IQR: 0·694 – 0·728)

**Fig. 6:**
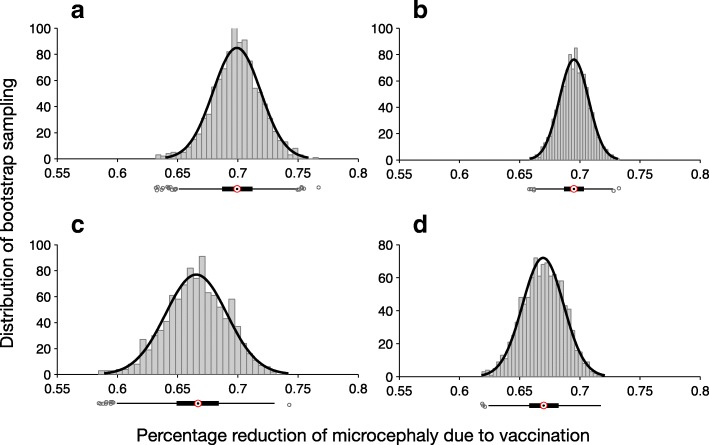
Distribution of percentage reduction of microcephaly obtained using bootstrap method, with *R*_0_ =2.8. Subplots correspond to the scenarios without pre-existing immunity (**a**, **b**), and with an average of 8% pre-existing immunity (**c**, **d**) in the population. The relative transmissibility of asymptomatic infection was set to 10%(**a**, **c**) and 90% (**b**, **d**). The median percentage reduction is (**a**) 0·699 (IQR: 0·687 – 0·712); (**b**) 0·695 (IQR: 0·687-0·704); (**c**) 0·666 (IQR: 0·649 – 0·683); (**d**) 0·670 (IQR: 0·658 – 0·682).

We used the cumulative number of fetal microcephaly cases following ZIKV infection during pregnancy at the end of each simulation in the absence and presence of vaccination. Percentage reduction of microcephaly due to vaccination was calculated using 2000 bootstrap replications. In all scenarios investigated for vaccine cost-effectiveness, the median percentage reduction of microcephaly exceeded 64% (Figs. 5 and 6), suggesting that a vaccine with protection efficacy as low as 60% could significantly reduce the incidence of microcephaly.

## Discussion

In this study, we evaluated the cost-effectiveness of a Zika vaccine candidate from a government perspective under a number of plausible scenarios. Using an agent-based model of ZIKV transmission dynamics, we determined the VCPI for each scenario, below which vaccination was cost-saving (when ICER values were negative) and highly cost-effective (when ICER values were positive, below the threshold of willingness-to-pay). Our analysis was based on using direct medical cost estimates associated with the treatment of symptomatic Zika infection, GBS cases, and long-term neurological sequelae caused by microcephaly condition. The results show that, in a population setting with similar characteristics to Colombia, targeted vaccination of women of reproductive age would be cost-saving in an outbreak response if VCPI was sufficiently low (i.e., scenario dependent), and cost-effective for a wide range of VCPI values between thresholds of one and three times per capita GDP. Although the likelihood of cost-effectiveness was shown to be sensitive to willingness-to-pay and vaccination costs, the largest range of VCPI for cost-effectiveness corresponded to scenarios in which the population is fully susceptible or the effect of other interventions to blunt ZIKV transmission is relatively low. However, non-pharmaceutical measures (including vector control programs), increased access to contraception [33], and pre-existing herd effects as a result of naturally acquired immunity in previous outbreaks could decrease the range of VCPI for cost-effectiveness, requiring a significantly higher willingness-to-pay for vaccination to prove cost-effective. In all scenarios, vaccination with an individual-level protection efficacy sampled in the range 60% to 90% resulted in a median reduction of microcephaly that exceeded 64% compared with no vaccination.

To our knowledge, this study presents the first cost-effectiveness analysis of a Zika vaccine candidate. We performed cost-effectiveness analysis using an individual-level stochastic approach and employed a bootstrap sampling method, which account for parameter uncertainty. A key strength of our modeling approach is that, unlike state-transition and static models, it inherently takes into account the indirect protection effects of naturally acquired and vaccine-induced immunity in the population. However, this study has several limitations arising from the lack of data and evidence. First and foremost is the lack of vaccine efficacy data in humans. While Zika virus challenge in rhesus monkeys has shown a high level of neutralizing antibodies for complete protection in a number of vaccine platforms [34], such information is currently unavailable for humans. The efficacy data can also provide information on the number of vaccine doses required, which would affect the vaccination costs per individual. In the absence of such information, we considered a single dose of vaccine with protection efficacy of 60% to 90%. We also assumed that the risk of microcephaly is independent of vaccine-induced immunity in a vaccinated pregnant woman if infection occured. In the absence of pre-existing immunity, clinical and epidemiological studies [7, 8] indicate that a significant portion (up to 80%) of ZIKV-infected individuals experience asymptomatic infection without presenting clinical symptoms. We assumed that vaccine-induced immunity further reduces the chance of clinical manifestation (if infection occurred), and therefore considered infection following vaccination to be asymptomatic. Validation of this assumption requires efficacy data from clinical trials, which are currently lacking. However, in terms of costs associated with microcephaly (which dominate), we expect our results of cost-effectiveness analysis to hold because we did not alter the risk of microcephaly in the presence of vaccine-induced immunity in pregnant women. Without the outcomes of clinical trials, our model did not consider the possible adverse side effects of vaccination and their associated costs. Although, other neurological disorders have been reported in association with ZIKV infection (including encephalitis, meningoencephalitis, myelitis, and optical neuritis) [35], we considered only microcephaly and GBS outcomes. In the context of cost-effectiveness analysis from a government perspective, our analysis excluded indirect costs such as loss of productivity and earnings in families inflicted by microcephaly and GBS, yet we understand that the lifetime indirect costs related to the care of children with microcephaly could be substantial [30].

In the model presented here, we considered individual interactions only for the implementation of sexual transmission. Due to the lack of individual movement data, our model does not include mobility patterns, which may influence the level of exposure to mosquito bites. We also note that the risk of sexual transmission may continue for several days or weeks following recovery from infection [36]. However, due to the uncertainty of this period at the individual level [36], we made a simplifying assumption of considering the risk of sexual transmission only during the infectious period. Despite these limitations that merit further investigation, our results suggest that a Zika vaccine has the potential to significantly reduce the health and economic burden of ZIKV infection in at-risk populations. In addition to informing policymakers with cost-effective scenarios of vaccination and its potential for outbreak containment, this study presents a comprehensive modeling approach that can be used to evaluate cost-effectiveness in other population settings and provide more accurate estimates as vaccine-specific data become available. Similar to other flavivirus vaccines such as dengue [32], understanding the effectiveness and health economics of a Zika vaccine is an important research priority, especially in the context of populations where ZIKV vector carriers (e.g., *Aedes aegypti*) are endemic.

## Conclusions

Our study suggests that a Zika vaccine with protection efficacy as low as 60% could significantly reduce the incidence of microcephaly. Vaccinating women of reproductive age was shown to be highly cost-effective for a large range of vaccination costs per individual with the threshold of willingness-to-pay corresponding to the average per capita GDP of Colombia between 2013 and 2017. Efficacy data from clinical trials and number of vaccine doses will be important requirements in future studies to refine our estimates and to conduct similar studies in other at-risk populations.

## Additional file

Additional file 1: Details of the model and its analysis with additional simulation results. (PDF 3785 kb)

## Abbreviations

GBS: Guillain**–**Barré Syndrome
ICER: Incremental cost-effectiveness ratio
VCPI: Vaccination costs per individual
WHO: World Health Organization
ZIKV: Zika virus
