# Supplementary material for: Cost-effectiveness of a potential Zika vaccine candidate: a case study for Colombia
Source: BMC Med. 2018 Jul 3;16:100. doi: 10.1186/s12916-018-1091-x (PMC6029035; doi:10.1186/s12916-018-1091-x)
Supplement: Supplementary file 1 — Details of the model and its analysis with additional simulation results. (PDF 3785 kb) [file 12916_2018_1091_MOESM1_ESM.pdf]

Cost-Effectiveness of a Potential Zika Vaccine Candidate: A Case Study for Colombia

Affan Shoukat, Thomas Vilches, Seyed M. Moghadas

This supplementary material provides further details of the model and additional simulation results supporting the analysis and discussion presented in the main text.

The Model

We extended an agent-based model of ZIKV transmission dynamics [1] to include disease outcomes and vaccination. The model simulates the spread of ZIKV infection between humans and mosquitoes, as well as through sexual interaction. The distributions of age and sex were set to those of Colombia (Figure S1), with a scaled-down population 10,000 individuals. The ratio of mosquito population size to human population size was set to 5 and 10 for $R_0 = 2.2$ and $R_0 = 2.8$, respectively. ZIKV transmission from mosquitos to humans (or vice versa) occurred as a result of rejection sampling-based (Bernoulli) trials, where the chance of successful transmission is defined by a probability distribution. This probability was calculated at the time of mosquito bite by $P_{\text{infection}} = 1 - (1 - \beta)^N$ where β is the baseline probability, calibrated to a given reproduction number, and N is the number of bites of a single mosquito to an infectious individual. Sexual transmission of ZIKV was implemented for individuals older than 15 years of age and in a monogamous context. The frequency of sexual encounters for partnered individuals was sampled from a distribution (Tables S1 and S2) derived from a national probability sample among adult men and women [2,3]. For an individual in the age group a_i, the partner was selected from the age group $a_i \pm 5$ years of age.

Table S1. Age-dependent probability matrix of sexual encounters for males.

	Weekly frequency of sexual encounters for males					
Age groups	0	1	2	3	4	5
15 – 24	0.167	0.167	0.229	0.229	0.104	0.104
25 – 29	0.109	0.463	0.1855	0.1855	0.0295	0.0275
30 – 39	0.201	0.473	0.134	0.134	0.029	0.029
40 – 49	0.254	0.51	0.0995	0.0995	0.0185	0.0185
50 – 50	0.456	0.383	0.075	0.075	0.0055	0.0055
60 – 69	0.551	0.354	0.0475	0.0475	0	0
70+	0.784	0.15	0.029	0.029	0.004	0.004

Table S2. Age-dependent probability matrix of sexual encounters for females.

	Weekly frequency of sexual encounters for females					
Age groups	0	1	2	3	4	5
15 – 24	0.265	0.147	0.1765	0.1765	0.1175	0.1175
25 – 29	0.151	0.477	0.176	0.176	0.01	0.01
30 – 39	0.228	0.502	0.1095	0.1095	0.0255	0.0255
40 – 49	0.298	0.466	0.104	0.104	0.0135	0.0145
50 – 50	0.457	0.362	0.0845	0.0845	0.0055	0.0065
60 – 69	0.579	0.359	0.031	0.031	0	0
70+	0.789	0.183	0.007	0.007	0.007	0.007

Infected individuals with ZIKV entered an intrinsic incubation period (IIP) before becoming infectious. This period was sampled for each individual from its associated distribution (Table 1 of the main text). After the IIP has elapsed, a fraction (sampled between 40%-80%) of infected individuals entered asymptomatic infection without developing clinical symptoms, and the remaining fraction developed clinical symptoms. We assumed that recovered individuals from either asymptomatic or symptomatic infection are immune against reinfection in the same epidemic season. For infected mosquitoes, we considered an extrinsic incubation period (EIP), which was sampled from its associated distribution (Table 1 of the main text). Once EIP has elapsed, infected mosquitoes become infectious and remain infectious for their remaining lifetime. Mosquito lifespan was determined by a hazard function given by [4]:

$$H(t) = \frac{ae^{bt}}{1 + \frac{as}{b}(e^{bt} - 1)}$$

We developed a discretized distribution generated by hazard and survival functions, and sampled mosquitoes lifetimes for both high temperature (a = 0·0018, b = 0·3228, and s = 2·1460) and low temperature (a = 0·0018, b = 0·8496, and s = 4·2920) seasons, with average lifespan of 19·6 and 11·2 days [1], respectively.

The mosquito bites were implemented as a Poisson process, with an average of 1 bite every 2 days. Previous studies [5,6] used estimates of 0.33 – 1 for biting rate with an average of 0.5. These estimates correspond to a range of one bite per day (rate=1) to one bite every 3 days (rate=0.33) on average. For the average of 0.5 (1 bite every 2 days), we considered the half-life of a single mosquito as the mean of a Poisson distribution, from which the number of bites was sampled, and randomly distributed over the mosquito lifetime, with a maximum of 1 bite per day.

Assumptions and Dynamics

Currently, quantification of several parameters associated with ZIKV infection is lacking. We therefore simulated the model with plausible values (ranges) for these parameters [1]. For the relative transmissibility of asymptomatic infection compared with symptomatic infection, we considered both low (10%) and high (90%), which was implemented as a reduction factor for ZIKV transmission from asymptomatic cases. We also assumed reduction factors of 10% and 50% for ZIKV transmission from symptomatic infection, to account for decreased mobility and lower exposure to mosquito bites through full clothing, mosquito repellents, or possible isolation during symptomatic infection. These reduction factors were implemented probabilistically.

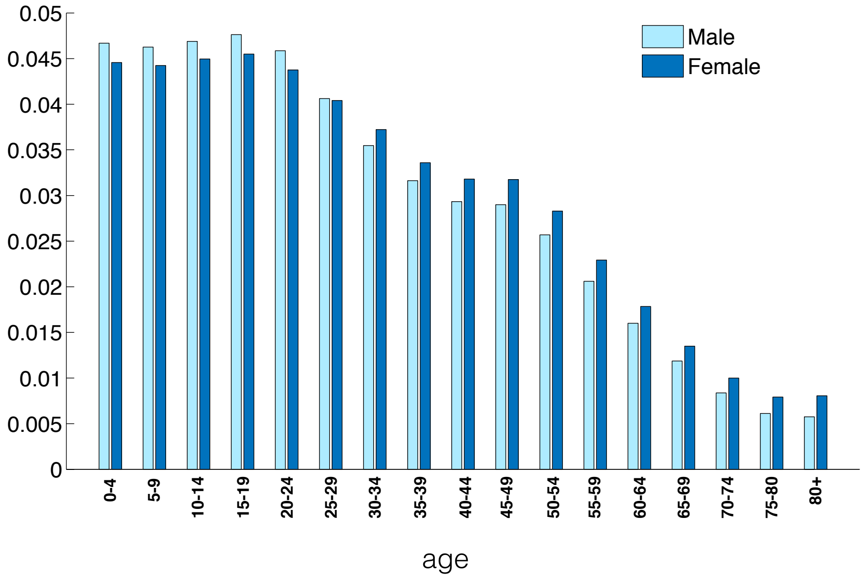

Figure S1. Age-sex distribution of the population of Colombia derived from census data [7].

Vaccination was implemented according to the WHO/UNICEF ZIKV vaccine target product profile [8], prioritizing women of reproductive age (WRA) and pregnant women. We assumed a vaccination coverage of 60% for non-pregnant WRA. For pregnant women, this coverage was increased to 80%. We also considered the inclusion of other individuals in the population between 9 and 60 years of age for vaccination with 10% coverage. The primary objective of this targeted vaccination is the prevention of prenatal ZIKV infection that may occur through mosquito bites or sexual transmission. In the absence of efficacy data, we assumed a range of 60% – 90% vaccine efficacy in preventing ZIKV infection using a single dose of vaccine. We assumed that vaccine-induced immunity does not change the risk of microcephaly if a vaccinated pregnant woman was infected with ZIKV. We also assumed that vaccine-induced immunity will prevent the development of clinical symptoms, and vaccinated individuals experienced asymptomatic infection (if infection occurs).

The total number of pregnant women was calculated based on the age distribution of population in each simulation (Figure S2). We used the fertility rate of 1.9/1000 women, and an estimated abortion rate of 12% for WRA [9]. The probability of birth for 9 months was assumed 0·75, and the probability of abortion for 2 months was assumed 0·167. Ignoring the fetal loss, the number of pregnant women at any point of time for each simulation was calculated by [10]:

$$\text{number of pregnant women} = \left(\frac{\text{nWRA}}{1000}\right)(\text{fertility rate} \times 0.75 + \text{abortion rate} \times 0.167)$$

where nWRA is the number of women of reproductive age. The number of pregnant women in 5-year age groups (between 15 and 49 years of age) was distributed according to the age distribution of pregnancy [11]. We considered trimesters to implement their risk of microcephaly in the model (Table 1 of the main text). At the onset of each simulation, the trimester for each pregnant woman was randomly selected according to the respective distributions [11]. The beginning of the first trimester was set for newly pregnant women during simulations. Microcephaly occurred according to the risk associated with infection in each trimester, which was implemented at the time of infection. Infants with microcephaly who survived the first year of life (with a probability of 79·8% [12]) had a reduced life expectancy, with an average lifetime of 35 years [13]. Life expectancy was sampled for each infant with microcephaly, and was used for calculation of DALY with the associated disability weights and direct medical costs.

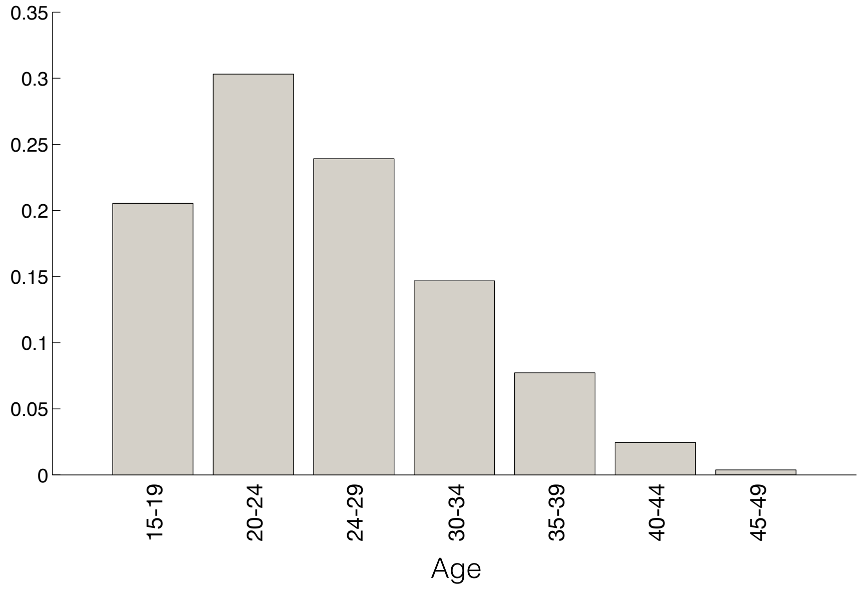

Figure S2. Distribution of pregnancy among women of reproductive age.

Disease Incidence

Reproduction number R_0=2·2

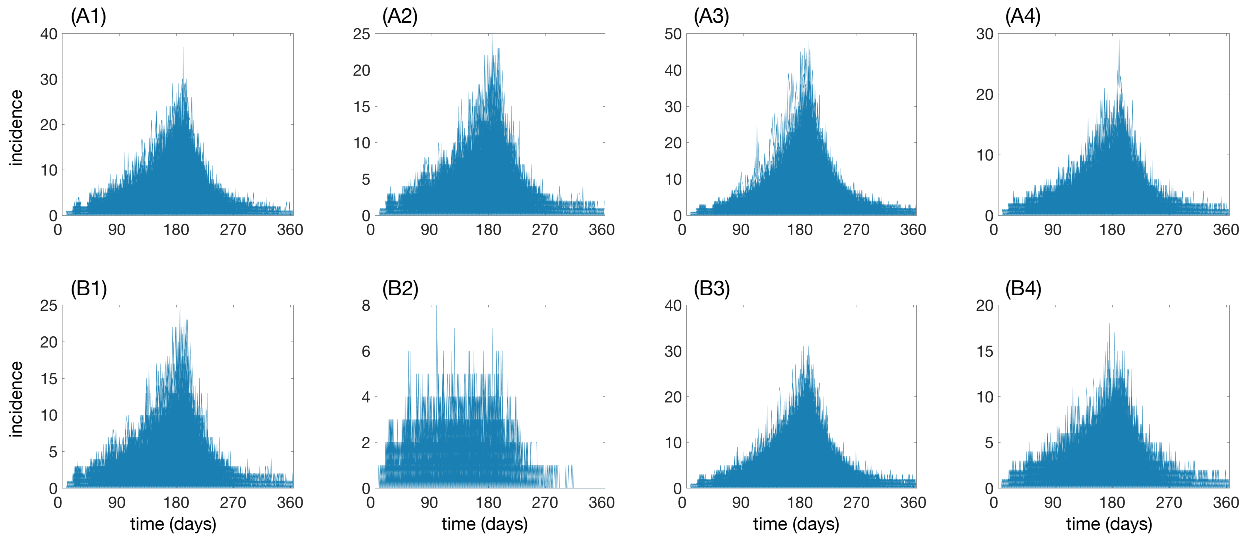

Figure S3. Incidence of infection for 5000 independent realizations without vaccination in the absence of herd immunity (A1-A4) and in the presence of 8% herd immunity (B1-B4) in the population. Simulations were run considering the relative transmissibility of asymptomatic infection and the reduction of transmission by symptomatic infection to be respectively: 0.1 and 0.1 (A1,B1); 0.1 and 0.5 (A2,B2); 0.9 and 0.1 (A3,B3); 0.9 and 0.5 (A4,B4).

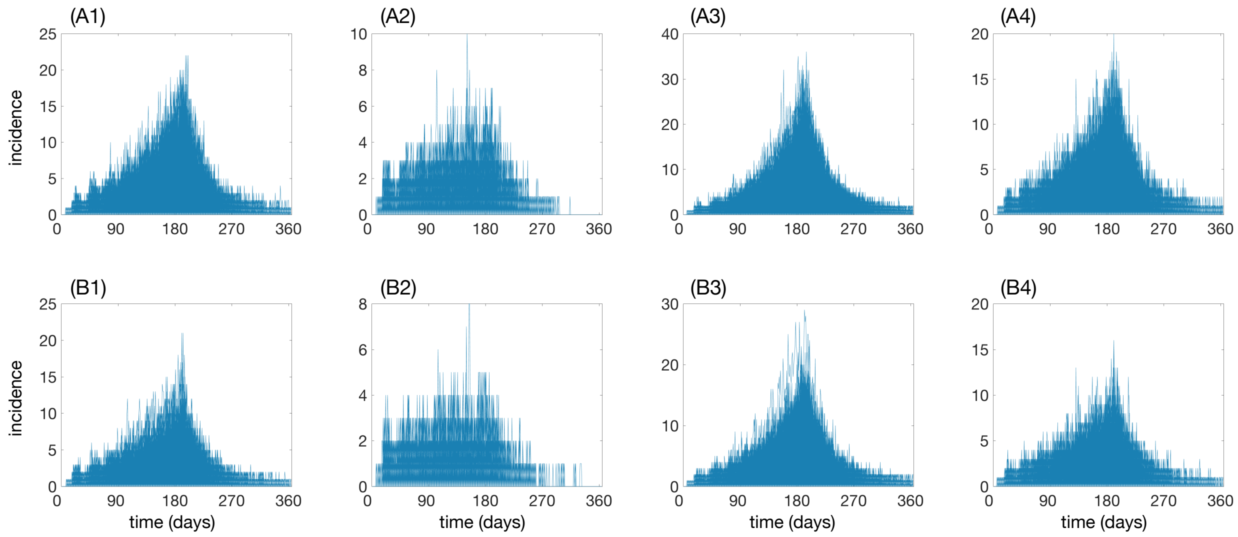

Figure S4. Incidence of infection for 5000 independent realizations with vaccination in the absence of herd immunity (A1-A4) and in the presence of 8% herd immunity (B1-B4) in the population. Simulations were run considering the relative transmissibility of asymptomatic infection and the reduction of transmission by symptomatic infection to be respectively: 0.1 and 0.1 (A1,B1); 0.1 and 0.5 (A2,B2); 0.9 and 0.1 (A3,B3); 0.9 and 0.5 (A4,B4).

Reproduction number R_0=2·8

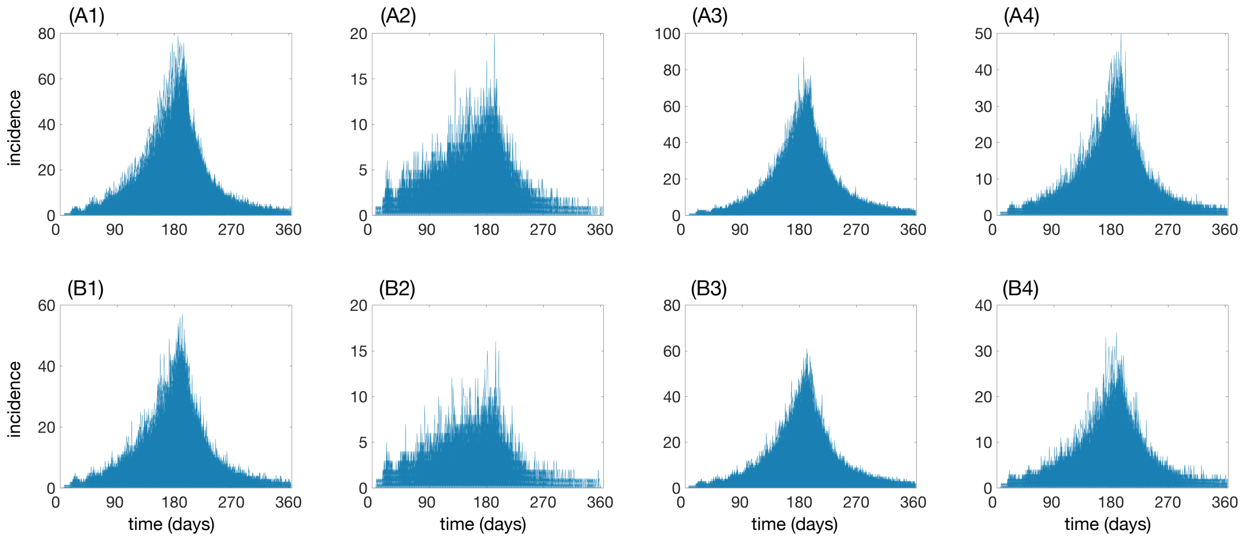

Figure S5. Incidence of infection for 5000 independent realizations without vaccination in the absence of herd immunity (A1-A4) and in the presence of 8% herd immunity (B1-B4) in the population. Simulations were run considering the relative transmissibility of asymptomatic infection and the reduction of transmission by symptomatic infection to be respectively: 0.1 and 0.1 (A1,B1); 0.1 and 0.5 (A2,B2); 0.9 and 0.1 (A3,B3); 0.9 and 0.5 (A4,B4).

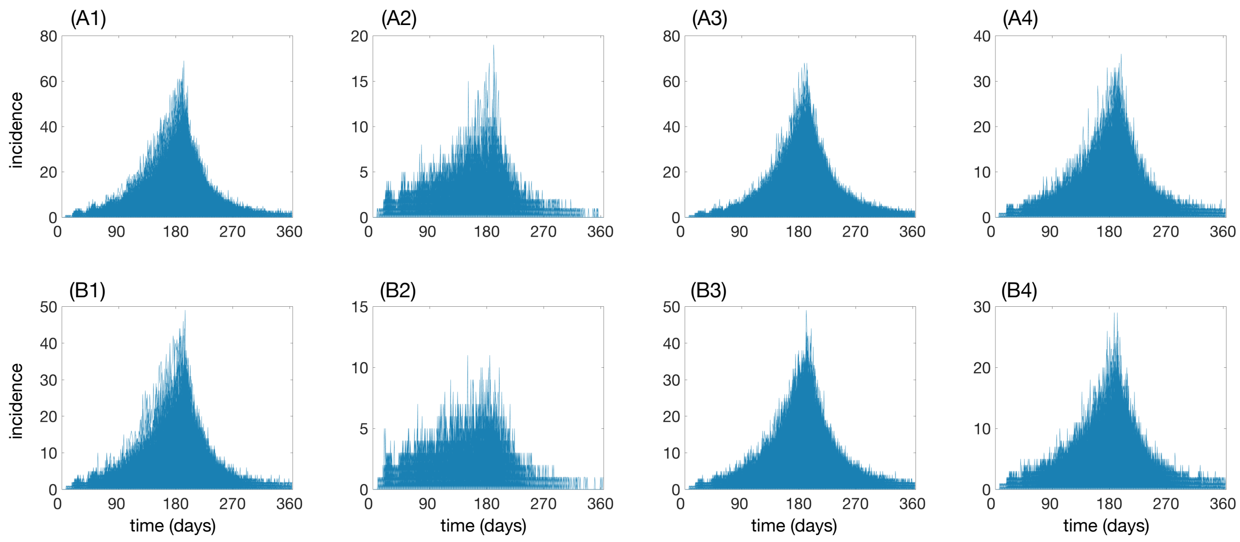

Figure S6. Incidence of infection for 5000 independent realizations with vaccination in the absence of herd immunity (A1-A4) and in the presence of 8% herd immunity (B1-B4) in the population. Simulations were run considering the relative transmissibility of asymptomatic infection and the reduction of transmission by symptomatic infection to be respectively: 0.1 and 0.1 (A1,B1); 0.1 and 0.5 (A2,B2); 0.9 and 0.1 (A3,B3); 0.9 and 0.5 (A4,B4).

Additional Simulation Results for Vaccine Cost-effectiveness

R_0=2·2 with 50% reduction of ZIKV transmission from symptomatic cases

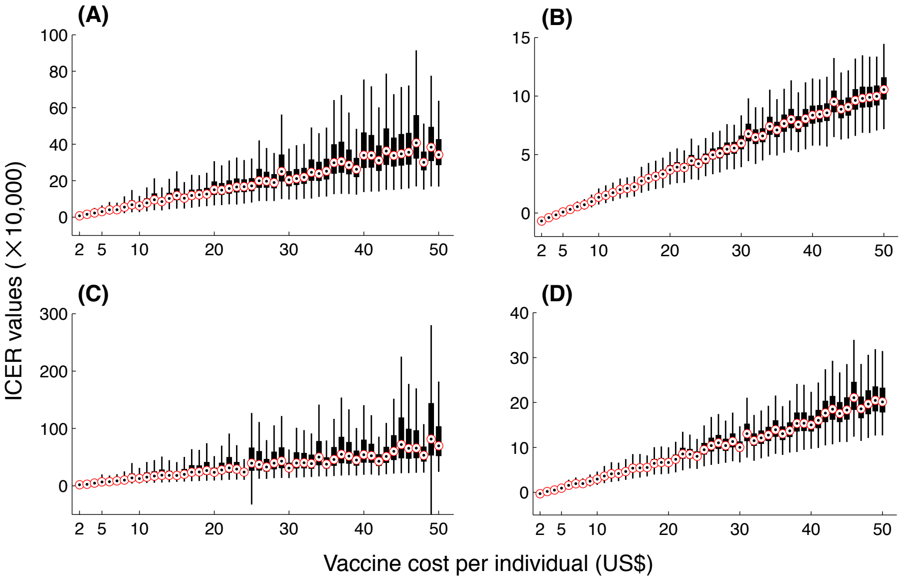

Figure S7. ICER values obtained using bootstrap method for a range of VCPI, when R_0=2·2 and the reduction of ZIKV transmission from symptomatic cases was set to 50%. Subplots correspond to the scenarios without pre-existing immunity (A,B), and with an average of 8% pre-existing immunity (C,D) in the population. The relative transmissibility of asymptomatic infection was 10% (A,C) and 90% (B,D).

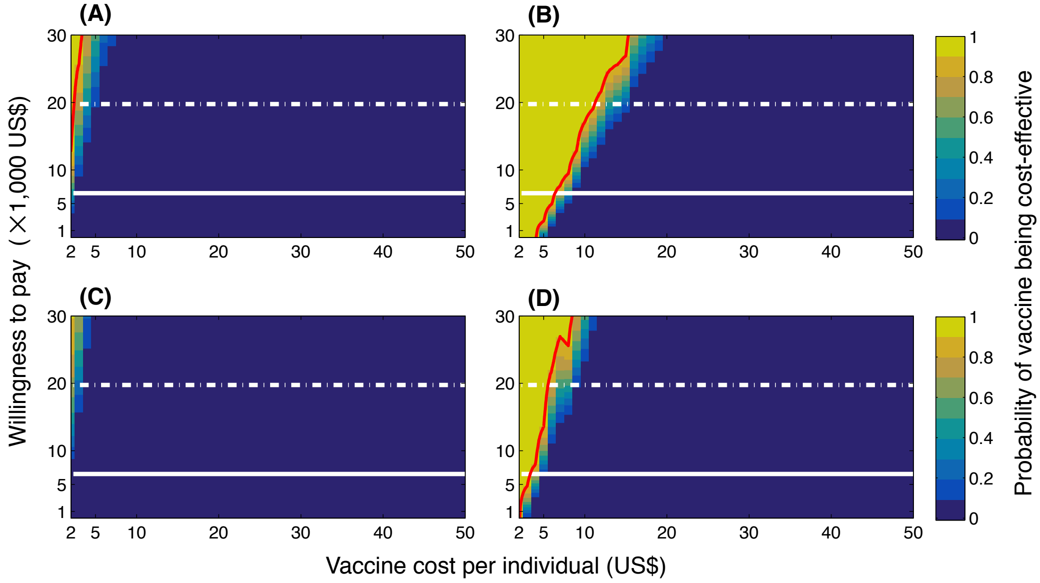

Figure S8. Probabilities of vaccine being cost-effective for a range of VCPI and willingness-to-pay, when R_0=2·2 and the reduction of ZIKV transmission from symptomatic cases was set to

50%. Subplots correspond to the scenarios without pre-existing immunity (A,B), and with an average of 8% pre-existing immunity (C,D) in the population. The relative transmissibility of asymptomatic infection was 10% (A,C) and 90% (B,D). Solid line represents the willingness-to-pay threshold corresponding to the average of per capita GDP of Colombia between 2013 and 2017. Dashed line represents three times the average of per capita GDP of Colombia. The red curve represents the 90% probability of vaccine being cost-effective for a given VCPI.

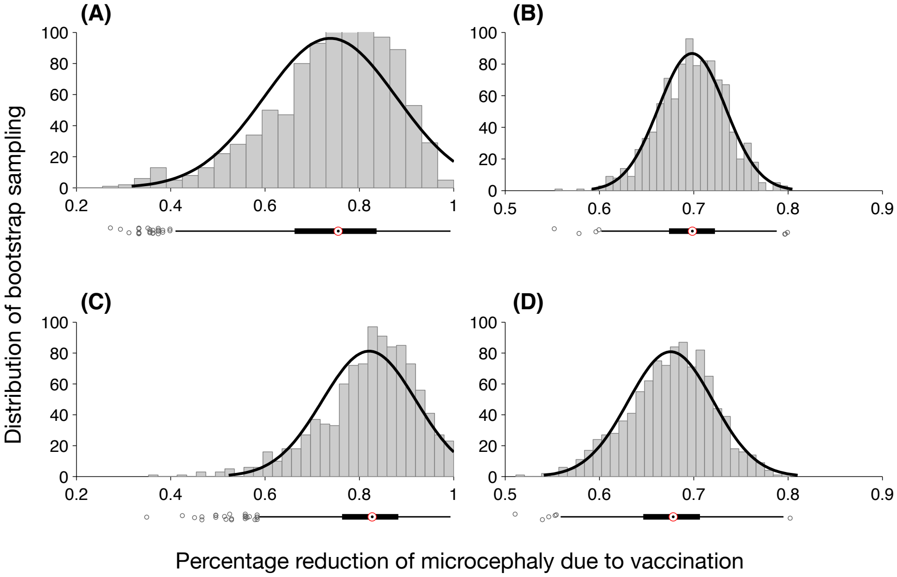

Figure S9. Distribution of percentage reduction of microcephaly obtained using bootstrap method, when R_0=2·2 and the reduction of ZIKV transmission from symptomatic cases was set to 50%. Subplots correspond to the scenarios without pre-existing immunity (A,B), and with an average of 8% pre-existing immunity (C,D) in the population. The relative transmissibility of asymptomatic infection was 10% (A,C) and 90% (B,D). The median percentage reduction is (A) 0·667 (IQR: 0·760 – 0·842); (B) 0·699 (IQR: 0·674 – 0·723); (C) 0·833 (IQR: 0·767 – 0·889); (D) 0·679 (IQR: 0·647 – 0·707).

R_0=2·8 with 50% reduction of ZIKV transmission from symptomatic cases

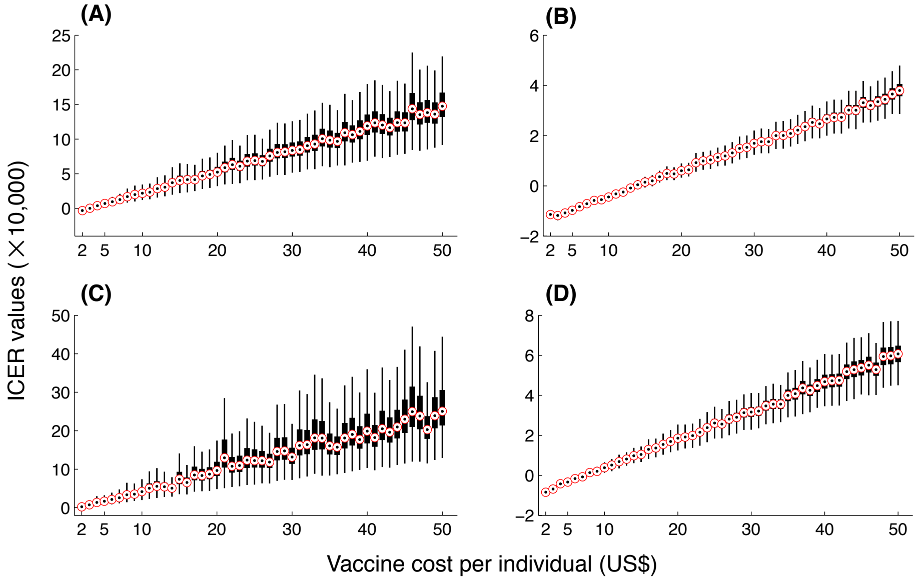

Figure S10. ICER values obtained using bootstrap method for a range of VCPI, when R_0=2·8 and the reduction of ZIKV transmission from symptomatic cases was set to 50%. Subplots correspond to the scenarios without pre-existing immunity (A,B), and with an average of 8% pre-existing immunity (C,D) in the population. The relative transmissibility of asymptomatic infection was 10% (A,C) and 90% (B,D).

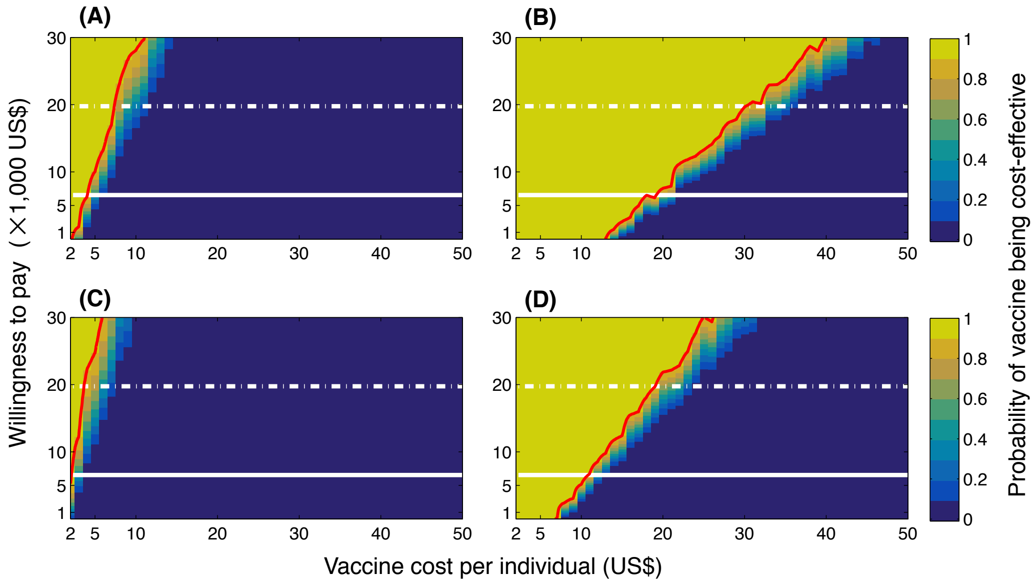

Figure S11. Probabilities of vaccine being cost-effective for a range of VCPI and willingness-to-pay, when R_0=2·8 and the reduction of ZIKV transmission from symptomatic cases was set to

50%. Subplots correspond to the scenarios without pre-existing immunity (A,B), and with an average of 8% pre-existing immunity (C,D) in the population. The relative transmissibility of asymptomatic infection was 10% (A,C) and 90% (B,D). Solid line represents the willingness-to-pay threshold corresponding to the average of per capita GDP of Colombia between 2013 and 2017. Dashed line represents three times the average of per capita GDP of Colombia. The red curve represents the 90% probability of vaccine being cost-effective for a given VCPI.

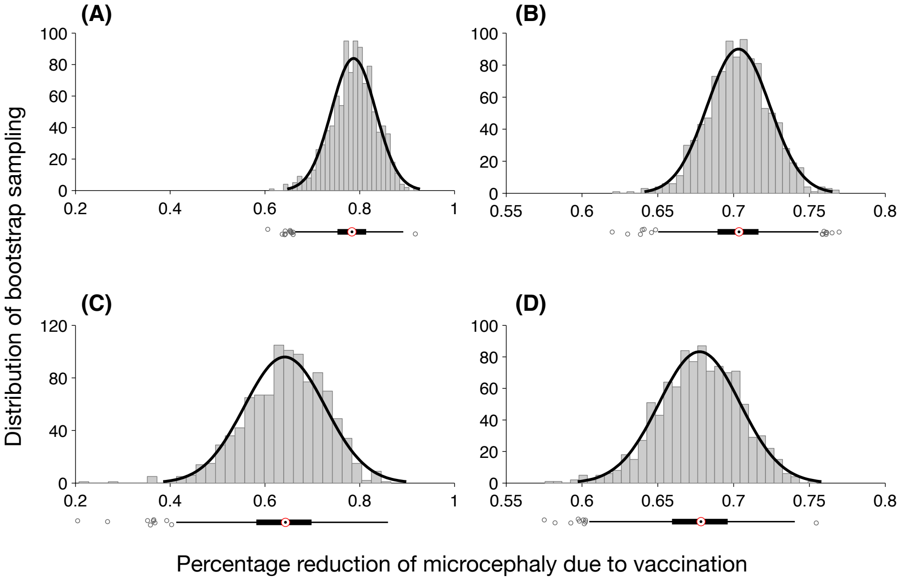

Figure S12. Distribution of percentage reduction of microcephaly obtained using bootstrap method, when R_0=2·8 and the reduction of ZIKV transmission from symptomatic cases was set to 50%. Subplots correspond to the scenarios without pre-existing immunity (A,B), and with an average of 8% pre-existing immunity (C,D) in the population. The relative transmissibility of asymptomatic infection was 10% (A,C) and 90% (B,D). The median percentage reduction is (A) 0·789 (IQR: 0·758 – 0·819); (B) 0·704 (IQR: 0·690 – 0·717); (C) 0·649 (IQR: 0·587 – 0·704); (D) 0·679 (IQR: 0·660 – 0·696).

References

1. Moghadas SM, Shoukat A, Espindola AL, Pereira RS, Abdirizak F, Laskowski M, Viboud C, Chowell G. Asymptomatic transmission and the dynamics of Zika infection. Sci Rep. 2017 Jul 19;7(1):5829.
2. Reece, M., Herbenick, D., Schick, V., Sanders, S.A., Dodge, B., & Fortenberry, J.D. Sexual behaviors, relationships, and perceived health among adult men in the United States: Results from a national probability sample. J Sex Med. 7(s5), 291-304 (2010).
3. Herbenick, D., Reece, M., Schick, V., Sanders, S.A., Dodge, B., & Fortenberry, J.D. Sexual behaviors, relationships, and perceived health status among adult women in the United States: Results from a national probability sample. J Sex Med. 7(s5), 277-290 (2010).
4. Styer, L. M., Carey, J. R., Wang, J. L., Scott, T. W. Mosquitoes do senesce: departure from the paradigm of constant mortality. Am. J. Trop. Med. Hyg. 76, 111-117 (2007).
5. Trpis M, Hausermann W. Dispersal and other population parameters of Aedes aegypti in an African village and their possible significance in epidemiology of vector-borne diseases. Am J Trop Med hyg. 1986;35(6):1263-79.
6. Putnam JL, Scott TW. Blood-feeding behavior of dengue-2 virus-infected Aedes aegypti. Am J Trop Med hyg. 1995;52(3):225-7.
7. United Nations Statistics Division - Demographic and Social Statistics. http://unstats.un.org/unsd/demographic/products/dyb/dyb2.htm
8. WHO/UNICEF Zika Virus (ZIKV) Vaccine Target Product Profile (TPP): Vaccine to protect against congenital Zika syndrome for use during an emergency: http://www.who.int/immunization/research/development/WHO_UNICEF_Zikavac_TPP_Feb2017.pdf
9. Daniels JP. Tackling teenage pregnancy in Colombia. Lancet. 2015;385(9977):1495-6.
10. Estimating the Number of Pregnant Women in a Geographic Area: http://www.cdc.gov/reproductivehealth/emergency/pdfs/pregnacyestimatobrochure508.pdf
11. Estimation of the number of pregnant women in Colombia: https://www.minsalud.gov.co/sites/rid/Lists/BibliotecaDigital/RIDE/VS/ED/GCFI/1.2%20Estimaciones%20GestantesNacimientos.pdf
12. Nembhard WN, Waller DK, Sever LE, Canfield MA. Patterns of first-year survival among infants with selected congenital anomalies in Texas, 1995–1997. Teratology 2001; 64: 267–275.
13. Alfaro-Murillo JA, Parpia AS, Fitzpatrick MC, Tamagnan JA, Medlock J, Ndeffo-Mbah ML, Fish D, Ávila-Agüero ML, Marín R, Ko AI, Galvani AP. A cost-effectiveness tool for informing policies on Zika virus control. PLoS Neglected Trop Dis. 2016;10(5):e0004743.
